# Highly Hydrophilic and Lipophilic Derivatives of Bile Salts

**DOI:** 10.3390/ijms22136684

**Published:** 2021-06-22

**Authors:** M. Pilar Vázquez-Tato, Julio A. Seijas, Francisco Meijide, Francisco Fraga, Santiago de Frutos, Javier Miragaya, Juan Ventura Trillo, Aida Jover, Victor H. Soto, José Vázquez Tato

**Affiliations:** 1 Departamento de Química Orgánica, Facultad de Ciencias, Universidad de Santiago de Compostela, Avda. Alfonso X El Sabio s/n, 27002 Lugo, Spain; pilar.vazquez.tato@usc.es (M.P.V.-T.); julioa.seijas@usc.es (J.A.S.); 2 Departamento de Química Física, Facultad de Ciencias, Universidad de Santiago de Compostela, Avda. Alfonso X El Sabio s/n, 27002 Lugo, Spain; francisco.meijide@usc.es (F.M.); santiagodefrutos@hotmail.com (S.d.F.); javier.miragaya@hotmail.com (J.M.); juanventur.trillo@rai.usc.es (J.V.T.); aida.jover@usc.es (A.J.); 3 Departamento de Física Aplicada, Facultad de Ciencias, Universidad de Santiago de Compostela, Avda. Alfonso X El Sabio s/n, 27002 Lugo, Spain; francisco.fraga@usc.es; 4 Escuela de Química, Centro de Investigación en Electroquímica y Energía Química (CELEQ), Universidad de Costa Rica, San José 11501-2060, Costa Rica; victor.soto@ucr.ac.cr

**Keywords:** bile acids and salts, partition coefficient, isothermal titration calorimetry, hydrophilic-lipophilic balance, lipophilicity, demicellization thermodynamics

## Abstract

Lipophilicity of 15 derivatives of sodium cholate, defined by the octan-1-ol/water partition coefficient (*log P*), has been theoretically determined by the *Virtual* *log P* method. These derivatives bear highly hydrophobic or highly hydrophilic substituents at the C3 position of the steroid nucleus, being linked to it through an amide bond. The difference between the maximum value of *log P* and the minimum one is enlarged to 3.5. The partition coefficient and the critical micelle concentration (*cmc*) are tightly related by a double-logarithm relationship ($Virtual\log P=-\left(1.00\pm 0.09\right)\log\left(\frac{cmc}{\mathrm{mM}}\right)+\left(2.79\pm 0.09\right)$), meaning that the Gibbs free energies for the transfer of a bile anion from water to either a micelle or to octan-1-ol differ by a constant. The equation also means that *cmc* can be used as a measurement of lipophilicity. The demicellization of the aggregates formed by three derivatives of sodium cholate bearing bulky hydrophobic substituents has been studied by surface tension and isothermal titration calorimetry. Aggregation numbers, enthalpies, free energies, entropies, and heat capacities, Δ*C_P,demic_*, were obtained. Δ*C_P,demic_*, being positive, means that the interior of the aggregates is hydrophobic.

## 1. Introduction

Natural bile salts, having a rigid skeleton with a peculiar distribution of their polar and apolar regions in a back-belly way, are surface active agents that self-aggregate in water solutions [1]. This family of steroids, which are solubilizers and emulsifiers of cholesterol, lipids, fat-soluble vitamins, fatty acids, and monoglycerides in mammals, includes sodium cholate (NaC), sodium deoxycholate (NaDC), and their glyco- and tauro-derivatives as the most representative compounds. Although depending on experimental conditions, their aggregates are usually small in size (low aggregation numbers) [1,2,3], and the monomers are packed in a back-to-back way with the hydrophobic surface of the steroid towards the aggregate interior and the hydrophilic one facing the solvent [2,4]. The fraction of bound counterions is also small [2].

Lipophilicity is a central physicochemical component of ‘the rule of 5′ [5] proposed for the analysis of potential new therapeutic drugs [6], by predicting their absorption, distribution, metabolism, excretion, and toxicity of drug candidates. It is frequently measured as octan-1-ol/H_2_O partition coefficient, *log P* [7].

The partition coefficient has been used to express the lipophilicity of bile acids and their salts [8,9]. Roda et al. [8,9] have experimentally determined the *log P* values for natural bile acids and their taurine and glycine conjugates, showing that it depends on the number, position, and orientation of hydroxyl groups. This dependence is also demonstrated by the retention parameters of reverse-phase chromatography [10,11]. In general, there is a good correlation between retention of a compound in reverse-phase liquid chromatography and octan-1-ol/water partition coefficient [6,12]. Other chromatographic methods have also been used [13,14].

Chemical modifications of the functional groups yield bile acid derivatives with enhanced or diminished lipophilicity. Oxidation of hydroxyl groups to oxo groups leads to derivatives with less tendency for self-aggregation, less membranolytic activity [15], and a decrease in the solubilization power of cholesterol [16]. However, the change in *log P* values for those compounds is rather low [15]. Other organic functions also modify the *log P* values [17]. Therefore, it should be expected that a wider range of *log P* values could be achieved by linking either hydrophobic or hydrophilic groups to the steroid nucleus.

During the last decade, we mainly focused our attention on bile acid derivatives at the C3 position. For instance, hydrophobic substituents drastically reduce the critical aggregation concentration with respect to the unmodified bile salt [18,19] and lead to the formation of a rich range of ordered supramolecular structures, including fibers and tubules [19,20], which show a wide range of sizes [21]. In many cases, the behavior may be tuned by the response to temperature [22] and pH [23] or by the preparation of catanionic mixtures [24]. However, these papers were mainly devoted to the description of the obtained supramolecular structures and, in some cases, to the mechanisms involved in their formation, but thermodynamic parameters associated with the aggregation process near the critical micelle concentration, *cmc*, were not determined. Since the *cmc* is clearly dependent on the hydrophobic/hydrophilic character of the substituent, it should be expected that the lipophilicity of the molecule was modified similarly. This can influence the biological properties of a given derivative, as the work by Herraez et al. [25,26,27] has proven (see below). Other properties have been correlated to hydrophilicity/hydrophobicity of bile salts as equilibrium cholesterol-solubilizing capacities [10]. Vlahcevic et al. [28] have concluded that the cholesterol-mobilizing capacity of bile salts appears to be a function of their relative hydrophobicity. Previous examples suggest that new unexplored biological properties can be expected for modified bile acids and that they would depend on the *log P* values. One of the purposes of this paper is to highlight a bile acid series with a wide range of *log P* values.

**Figure 1:**
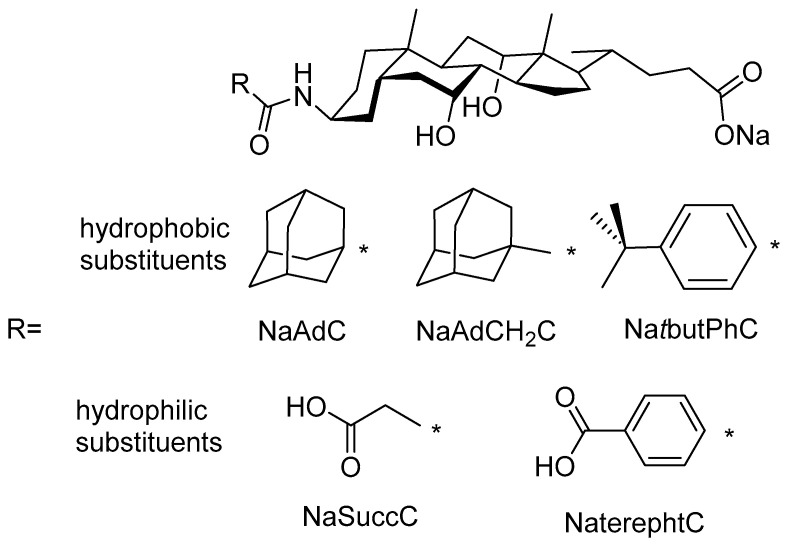
Structure of hydrophobic and hydrophilic derivatives of sodium cholate.

Thus, derivatives of cholic acid bearing highly hydrophobic or highly hydrophilic substituents at the C3 position are studied (Figure 1). It should be expected that previous derivatives, with bulky hydrophobic groups, have higher partition coefficients than natural bile acids. By contrast, modified bile acids with highly hydrophilic moieties such as succinoyl (NaSuccC) and terephthaloyl (NaterphtC) should lead to low *log P* values [25,26,29]. Partition coefficients are calculated theoretically and compared to *cmc* experimental values. The thermodynamics and aggregation numbers of the three derivatives bearing bulky hydrophobic groups have also been studied.

## 2. Results and Discussion

**Figure 2:**
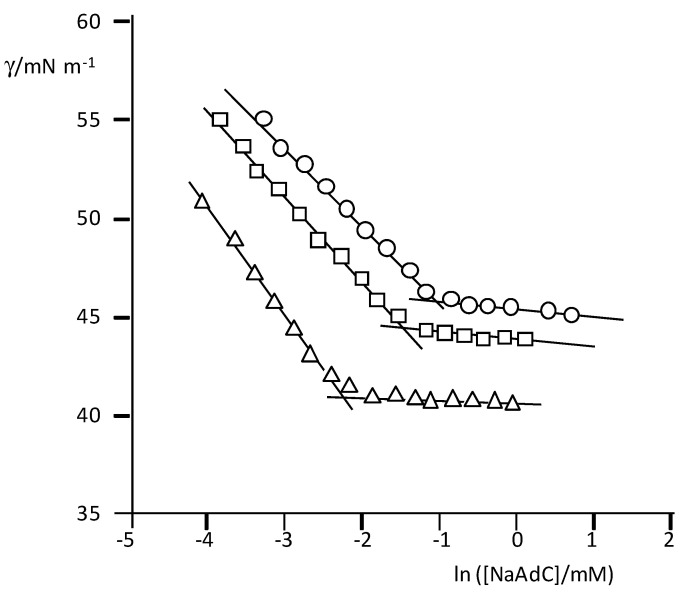

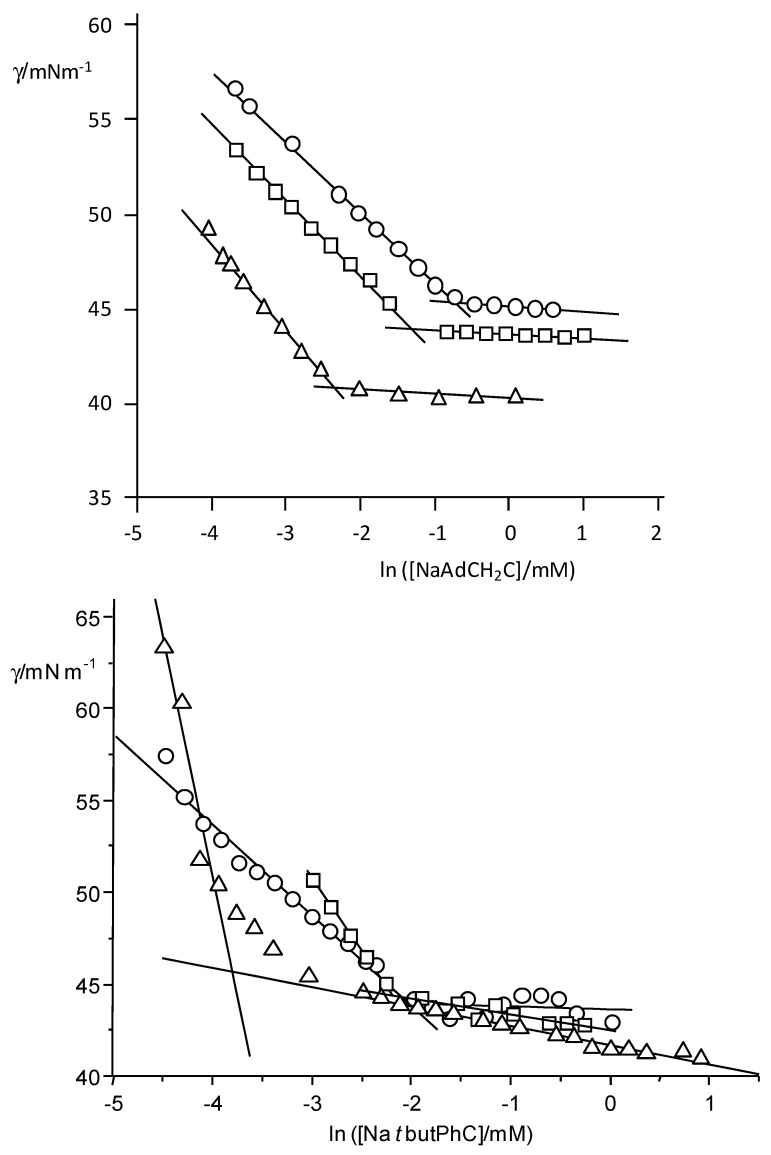
Plots of γ (mNm^−1^) vs. ln([surfactant]/mM) in carbonate/bicarbonate buffer with the indicated total concentrations; T = 25 °C. NaAdC (top): 0.050 (circles), 0.150 (squares), and 0.70 M (triangles). NaAdCH_2_C (middle): 0.0506 (circles), 0.150 (squares) and 0.70 M (triangles)Na*t*butPhC (bottom): 0.050 (circles), 0.150 (squares), and 1.00 M (triangles).

The results of the surface tension (γ) measurements for the three surfactants are plotted in Figure 2. The typical dependence of γ with the surfactant concentration is observed, and from the breaking point, the *cmc* values were obtained.

The *cmc’s* of the three anionic surfactants are influenced by the counter ion concentration in the aqueous medium. From this dependence, the degree of counterion binding (*β*) in micelles (Corrin–Harkins plot) may be obtained [30,31]. It may be written as

(1) $$\log cmc=A-\beta \log\left[Na^{+}\right]$$

**Figure 3:**
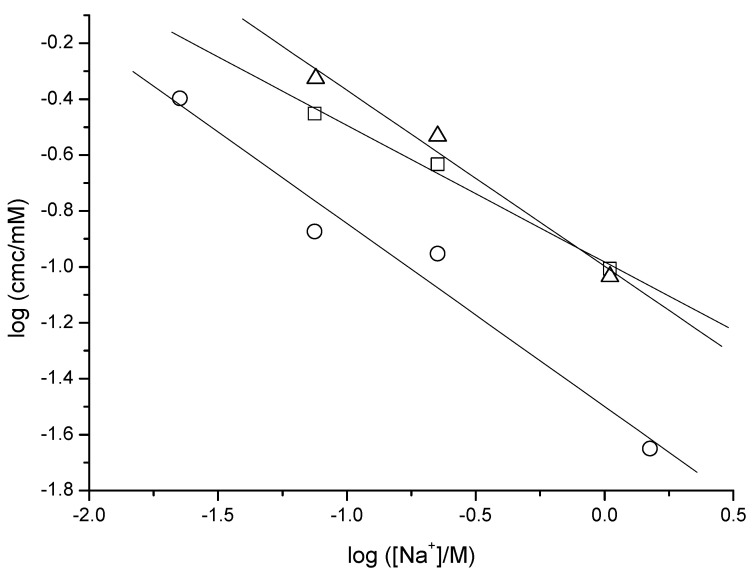
Dependence of *cmc* with the counterion concentration (Corrin–Harkins equation) for NaAdC (squares), NaAdCH_2_C (triangles), and Na*t*butPhC (circles).

The *β* values obtained from the slopes of the straight lines in Figure 3 are 0.49 (NaAdC), 0.63 (NaAdCH_2_C), and 0.65 (Na*t*butPhC), which are within the range (0.26–0.66) of published values for sodium alkanoates [32,33,34].

**Figure 4:**
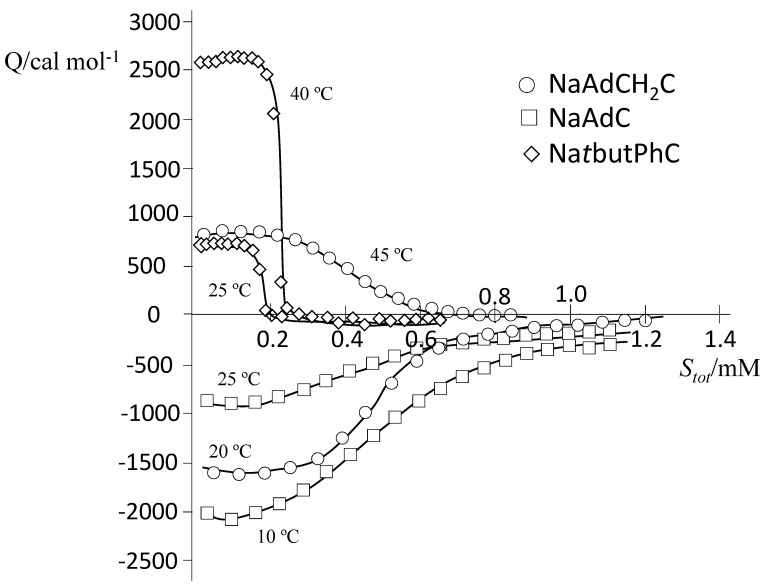
Representative ITC experiments in carbonate/bicarbonate buffer with a total concentration of 50.60 mM for NaAdC (squares, at 10 and 25 °C), NaAdCH_2_C (O, at 20 and 45 °C; and Na*t*butPhC (rhombuses, at 25 and 40 °C).

To fully characterize the cholate derivatives with attached bulky hydrophobic moieties, ITC experiments were carried out at temperatures in the range 10–45 °C. Figure 4 shows representative examples of ITC measurements for determining the thermodynamic parameters corresponding to the demicellization process of the aggregates of the three surfactants. In the range of temperatures studied, Δ*H_demic_* is negative at low temperatures and positive at high temperatures for NaAdCH_2_C and always negative for NaAdC (exothermic) and always positive for Na*t*butPhC (endothermic).

**Figure 5:**
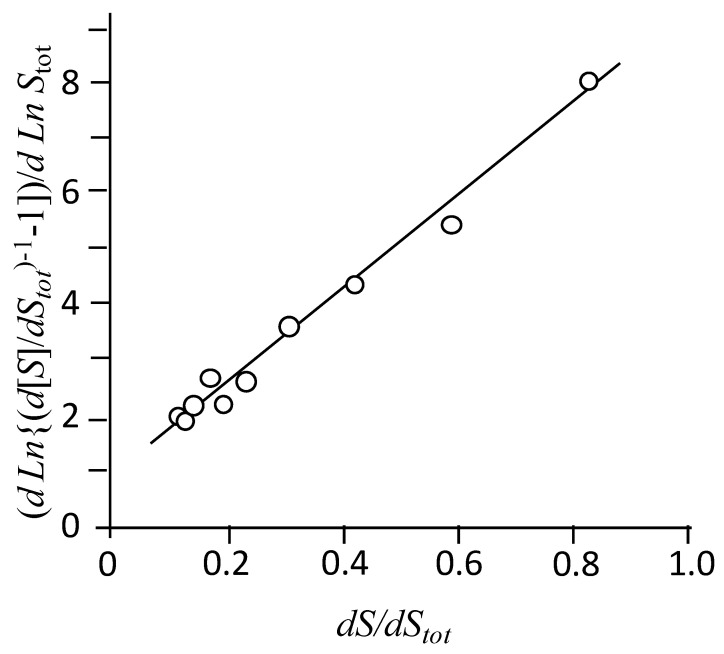
Determination of the aggregation number according to Equation (2). Concentration of injected NaAdCH_2_C 10.17 mM; total buffer concentration 50.60 mM, pH 10.35. T 45 °C.

Figure 5 shows an example of the calculation of the aggregation number for NaAdCH_2_C. In this case, *intercept* = 0.89, and *slope* = 8.43 (*r*^2^ = 0.986), from which a value of 10.5 was deduced for the aggregation number. Values at other temperatures and the thermodynamic magnitudes associated with the demicellization process are summarized in Table 1. The contribution of the term ${\left[Na^{+}\right]}^{\beta n}$ to Δ*G_demic_* is almost constant in the ranges of 3.9–4.2, 3.0–3.1, and 4.2–4.5 kJ mol^−1^ for NaAdCH_2_C, NaAdC, and Na*t*butPhC, respectively. It may be noticed that the entropy contribution is responsible for the association of these amphiphiles at all temperatures.

Although determined by different experimental techniques, the values of the *cmc’s* for the three surfactants at 25 °C are fully compatible with those obtained from surface tension.

Under normal conditions, aggregates of natural bile salts exhibit low aggregation numbers [1,2,3]. The values depend on experimental conditions as temperature, pH, or NaCl concentration. From ITC measurements, values in the ranges 4.7–6.1 for NaC [35] and 5–12.3 for NaDC [35,36] were obtained, in good agreement with those determined from other experimental techniques [2]. Olesen, Holm, and Westh have determined a value of 7.4 for sodium glycochenodeoxycholate at 37 °C [37], and Kroflič et al. [38] have obtained values of 5−6.7 (depending on experimental conditions) for 3-[(3-cholamidopropyl)-dimethylammonium]-1-propanesulfonate (CHAPS). Thus, the average values for NaAdC (6.5 ± 0.7) and NaAdCH_2_C (10.8 ± 1.5) are close to previous ones. However, the aggregation number (*n* = 44 ± 9) for Na*t*butPhC is far from all previous values suggesting that a different structure for the aggregates is probably formed. It has been demonstrated that Na*t*butPhC generates molecular tubes obeying a mechanism according to which initial spherical vesicles collapse into peanut-shaped structures that self-transform into tubules of small diameter. The association of these intermediate structures, parallel arranged, originates the final molecular tubes [19].

For the three hydrophobic derivatives, the demicellization enthalpy depends linearly on the temperature, with the slope being Δ*C_P,demic_* > 0. This means that the hydrophobic surface of monomers, being exposed to water, increases upon demicellization. This is in agreement with the back-to-back model and with the fact that transferring hydrophobic molecules from a nonpolar medium to water is always characterized by positive values of the heat capacity [39]. This is common for all ionic surfactants including natural bile salts [35,36]. In fact, the process has many facts in common with the dissolution of liquid alkanes into water [39,40,41].

For NaC and NaDC, Blume et al. [35] concluded that the total change in heat capacity is almost exclusively due to the change in hydrophobic hydration and estimated that the total change in exposed hydrophobic surface is rather small. However the ratio of the fluorescence intensities of the first and third vibronic peaks, *I*_1_/*I*_3_, of monomeric pyrene solubilized within NaC and NaDC aggregates suggests that the pyrene microenvironment is very apolar, in agreement with the fact that the fraction of pyrene in the interior of the small NaC and NaDC aggregates, which stay in contact with water, is only 4 and 0%, respectively [42]. These two observations suggest that pyrene is fully protected from the bulky solvent. Therefore, an underestimation of the actual hydrophobic surface of the steroid molecules not accessible to water cannot be ruled out.

The Δ*C_P,demic_* values for the adamantyl derivatives (335 and 300 J mol^−1^K^−1^ for NaAdC and NaAdCH_2_C, respectively) are between those for NaC (240–260 J mol^−1^K^−1^) and NaDC (340–360 J mol^−1^K^−1^) [35], suggesting that the effect of adding a bulky hydrophobic adamantyl group to the steroid nucleus is similar to the loss of the hydroxyl group at C7 in NaC. For CHAPS, values for Δ*C_P,demic_* around 210 J mol^−1^K^−1^ have been reported [38]. Nevertheless, for Na*t*butPhC, Δ*C_P,demic_* = 464 J mol^−1^K^−1^ is much higher than all previous values, suggesting a larger change in exposed hydrophobic surface to water after demicellization. The amazing *I*_1_/*I*_3_ ratio (1.55, at 25 °C) for Na*t*butPhC [19] indicates that pyrene is in a rather polar medium (in cyclohexane *I*_1_/*I*_3_ ~ 0.6 and in water, *I*_1_/*I*_3_ = 1.96) [43]. Thus, pyrene probably lies partially outside of the interior of an aggregate suggesting that the interaction probe-host is not strong enough to disturb the packed aggregates formed by this derivative. In comparison to the other surfactants, the behavior of Na*t*butPhC is obviously different.

The HLB concept has been repeatedly invoked in bile salt literature. Let us remember that, established on an empirical basis, HLB has been used in emulsion technology. As mentioned earlier, the coefficient of partition (*log P*) of a molecule between octan-1-ol and water [8] has been used to express the lipophilicity of bile acids and their salts [9]. Davies [44] proposed an equation which relates HLB with *P*, thus allowing for “an extrathermodynamic significance to each structural element in determining the molecule’s ability to function as a wetting agent, detergent, or defoamer” [7]. When the compound ionizes in the aqueous phase, it is common to use the distribution coefficient (log D) which is related to log P and the pK_a_ of the weak acid. However, it also depends on the bulky environment properties as pH [12]. On the other hand, Roda et al. [9] have shown that both the protonated and ionized forms of bile acids can distribute in octan-1-ol, and consequently, a partition coefficient for each form must be defined. The difference between both values is almost constant with an average value of 0.91 ± 0.09 **(***log P* units). Since the *cmc* involves the aggregation of the surfactant in its anion form and *log P* is related to that concentration, in what follows, the *log P* of the monoanion is considered.

Measurement of log *P* through synthesis of the compound and its subsequent experimental determination is time consuming and costly [45]. From their retrospective analysis, Tetko et al. [46] have suggested that the use of the predicted *log P* values, eliminating the need of experimental measurements, could reduce the cost of measurements for pharmaceutical companies. According to Daina et al. [47], “the experimental determination of *log P* remains a resource- and time-consuming procedure”, and for Tsopelas et al. [48], “partitioning experiments have rather low throughput”. Many methods for computing *log P* have been proposed [45]. However, these methods must be confident enough to obtain reliable values, thus requiring some experimental value as references for comparative purposes. For instance, equations only considering the number of carbon atoms and the number of hetero atoms [46] are not applicable to bile acids as it is necessary to distinguish between different isomers. The molecular lipophilicity potential (MLP) is shown to be sensitive to conformational effects and allows the calculation of *log P* [49]. Because of the reasons given below, this method was the one chosen to carry out the theoretical calculations of *log P*.

Table 2 shows the calculated values by the indicated methods together with the experimental values from Roda et al. [9] for natural unconjugated bile acids. The first two rows (NaC and NaHC) correspond to trihydroxy derivatives and the remaining rows to dihydroxy ones.

The Table shows that WLOGP, MLOGP, and SILICOS-IT do not distinguish between the isomers although they have different lipophilicity as demonstrated by reverse-phase chromatography [10,11] and the experimental log *P* values. We have checked this fact with other families of derivatives as mohydroxy and dihydroxy derivatives of adamantyl carboxylic acid. Therefore, these methods should be ruled out. On the hand, the summation $\sum {\left(\log P_{theory}-\log P_{exp}\right)}^{2}/n$ (where *n* = 6 is the number of bile salts) gives the average error for the whole derivatives. The values are shown in the last row of Table 2. According to Tsopelas et al. [48], predictions with residual equal or less to 0.5 are considered reliable; predictions with residuals between 0.5 and 1 are still acceptable; and predictions with residuals higher than 1 are not acceptable. Therefore, it may be concluded that *Virtual logP* is the most appropriate for the estimation of log P for bile acids. This may be a consequence that the molecular lipophilicity potential (MLP) is shown to be sensitive to conformational effects and allows the calculation of *log P* [49].

**Figure 6:**
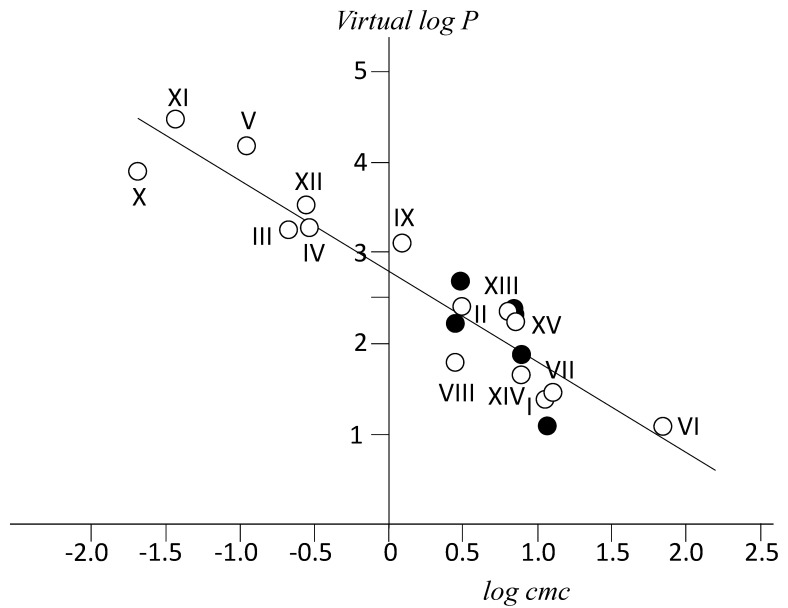
Calculated *Virtual log P* values vs. experimental *log* (*cmc/*mM).(Open circles) *cmc* values for I (NaC) and II (NaDC) from Roda et al. [50]; III (NaAdC), IV (NaAdCH2C), and V (NatbutPhC), this paper; VI (NaSuccC) and VII, NaterephtC [29]; VIII (sodium chenodeoxycholate, NaCDC) and IX (sodium obeticholate) [51]; X (NaAd(CH_2_)_2_C) and XI (NaNAPhDC) see Supplementary information; XII (sodium litocholate), *cmc* in [52]. Experimental values (black circles) by Roda et al. [9]: I (NaC), II (NaDC); VIII (NaCDC); XIII (sodium ursodeoxycholate); XIV (hyocholate); XV (hyodeoxycholate). Structures are given as Supplementary Materials.

Calculated virtual *log P* values for all previous derivatives are plotted against *log cmc* in Figure 6. This figure also includes natural bile salts and other derivatives (see legend of Figure 6) whose *cmc* values can be found in literature and the corresponding *log P* values have been calculated by us. The equation of the fitted straight line is

(2) $$Virtual\log P=-\left(1.00\pm 0.09\right)\log\left(\frac{cmc}{\mathrm{mM}}\right)+\left(2.79\pm 0.09\right)\;(r^{2}=0.948)$$

Previous equation allows for the estimation of either *log P* from the knowledge of *cmc* or vice versa. The first one is easily estimated theoretically, while the second one is the most common property determined experimentally for a new surfactant derivative. It can be noticed that the range of Δ*log P* values extends now to up 3.5 (*log P* units). NaterephtC and NaC, with structures which differ significantly, have very similar *cmc* values (11.6 and 13 mM respectively) as well as *log P* calculated ones. This supports the validity of the relationship.

From a simple analysis of mass action law applied to micelle formation, under the isodesmic model, the equilibrium constant for the association of a monomer to a micelle with (*j-*1) monomers to form a micelle with *j* monomers [53,54,55], *K_o_*, is associated with the inverse of the monomer concentration at *cmc*. That is to say, $\Delta G_{mic}^{0}=-RT\ln K_{o}$ for the transfer of a monomer from the bulk solution of a micelle of (*j-1*) monomers to form a new micelle with *j* monomers. On the other hand, the free energy of transfer of a solute from water to octan-1-ol is $\Delta G_{transfer}^{0}=-RT\ln P$ [47]. These relationships together with Equation (6) indicate that the transfer of a bile anion from water to either a micelle or to octan-1-ol differ by a constant. This is a consequence that both processes share the transfer of a hydrophobic moiety from an aqueous environment to a lipophilic one and suggests that the bile anions tend to be adsorbed at the octan-1-ol/water interface (with the hydrophilic face towards water) as happens at the air/water interface previously to micelle formation.

A nice example which illustrates the different biological behavior of bile salt derivatives with extreme *log P* values has been provided by Herraez et al. [25,26,27]. These authors investigated the ability of several bile acid derivatives to prevent OATP-mediated toxin uptake by the liver without affecting the uptake of natural bile acids. All the four investigated compounds (two bile acid dimers and two monomers—HSuccC and HAdC, the acid forms of the corresponding salts)—induced a dramatic inhibition in human OATP1B1-mediated [3H]-TCA uptake, but none of them were able to significantly inhibit human OATP1B3-mediated [3H]-TCA uptake. However, marked differences were observed in the ability of these compounds to inhibit human NTCP- and rat NTCP-mediated [3H]-TCA uptake. Thus, while the highly hydrophilic compound SuccC (*log P* = 1.00; named BALU-1 by Herraez et al.) had no effect on either human NTCP or rat NTCP, the highly hydrophobic AdC (*log P* = 3.23; named BALU-2 in those papers) had a strong inhibitory effect. The difference in *log P* of both compounds is as high as 2.2 units. Furthermore, it was demonstrated that liver uptake of *Phalloidin* through the transporter OATP1B1 is inhibited by NaSuccC, which, as indicated, does not inhibit the sodium-dependent bile acid transporter NTCP. As far as we know, this is the only biological study involving the new derivatives.

## 3. Experimental

Synthesis and chemical characterizations of NaAdC [56], NaAdCH_2_C [57], Na*t*butPhC [56], NaSuccC, and NaterephtC [29,58] have been published previously.

Surface tension measurements were carried out in a drop volume tensiometer TVT2 from Lauda. Temperature was kept constant at 25 °C by recirculating water from a PolyScience 9100 thermostat. Average surface tension values were obtained from four series of 3–6 drops each. Sets of solutions were prepared according to the step-by-step dilution-extraction method [59].

Calorimetric studies on the demicellization of the mentioned hydrophobic surfactants were carried out by isothermal titration calorimetry (ITC) in a MicroCal ITC200 titration calorimeter (Malvern Panalytical, Malvern, UK) at constant temperature. The heat exchange for each injection was calculated by the Origin program supplied by MicroCal, which accepts a fast mixing in the measuring cell during each injection from the syringe. In a typical experiment, 2 μL of a 4–10 mM (depending on the derivative) solution of the surfactant in a carbonate/bicarbonate buffer solution (with different total concentrations but identical ratio of components to obtain a pH value of 10.35) was injected into an aqueous solution with identical buffer concentration. This pH assures a complete ionization of the carboxylate head group.

Experimental titration curves were analyzed according to Olesen, Holm, and Westh [37]. The model assumes an equilibrium between surfactants in monomer state, *S*, and aggregates, *M_n_*, characterized by an aggregation number *n* and a micellization constant *K* (Equation (1)).

(3) Sn⇌Mn

(4) $$K=\left[M_{n}\right]/{\left[S\right]}^{n}$$

By taking into account that the monomer concentration is related to the total surfactant concentration by $S_{tot}=\left[S\right]+nK{\left[S\right]}^{n}$, Equation (2) is derived after several transformations.

(5) $$\frac{d\;ln\{{\left(d\left[S\right]/dS_{tot}\right)}^{-1}-1\}}{d\;lnS_{tot}}=\frac{n-1}{n}+\frac{{\left(n-1\right)}^{2}}{n}\frac{dS}{dS_{tot}}$$

The amount $dS/dS_{tot}$ is obtained from the calorimetric data as the quotient between the enthalpy change after adding a small amount of surfactant from the syringe into the measuring cell and the enthalpy of demicellization, $d\Delta H/\Delta H_{demic}$. The analysis of the experimental data according to Equation (2) affords the values of *n*, $\Delta H_{demic}$ and the involved enthalpy of dilution as optimization parameters. Finally, the equilibrium constant is obtained from *n* and *cmc*.

For ionic surfactants such as those studied in this paper, it is necessary to take into account the degree of counter-ion binding. This consideration implies [37,60]

(6) $$K_{mic}=K{\left[Na^{+}\right]}^{\beta n}$$

which allows the calculation of the free energy change associated with demicellization, Δ*G_demic_*, as

(7) $$\Delta G_{demic}=\left(\frac{RT}{n}\right)lnK_{mic}$$

For the calculation of log P values, the coordinates of the molecules were taken from the structures obtained from X-Ray crystallography ([61] and references therein).

Several computational methods have been published for the determination of log *P* [62]. In order to choose the best option for bile salts, the obtained theoretical values were compared with those experimentally available [9]. Internal self-coherence was considered as well (see below). The following methods were used: iLOGP [47], XLOGP3 [63], WLOGP [64], MLOGP [5,65,66], and SILICOS-IT [67]. The values were calculated using the web service from Swiss Institute of Bioinformatics SwissADME tools (Lausanne, Switzerland) [68] available at http://www.swissadme.ch/ (accessed on 21 June 2021), allowing for the computation of physicochemical descriptors as well as to predict ADME parameters. The virtual logP [49] is obtained by the molecular lipophilicity potential (MLP), which is calculated by projecting the Broto–Moreau lipophilicity atomic constants on the molecular surface. It was evaluated by VegaZZ software [69,70]. VegaZZ software can be downloaded free of charge at http://www.vegazz.net (accessed on 21 June 2021), which also provides the values according to Broto [71].

## 4. Conclusions

For all known bile salt derivatives, the heat capacity change upon demicellization is positive, indicating that the interior of the aggregates is hydrophobic in nature and that, the hydrophobic surface of each monomer, being exposed to water, increases upon demicellization. This suggests that the monomers are packed in a back-to-back way according to Small’s model. Extending the hydrophobic region of natural bile salts does not necessarily imply a significant increment of the aggregation number of micelles above cmc. However, in comparison to the other hydrophobic surfactants, the behavior of Na*t*butPhC is different.

Lipophilicity of bile acids can be easily modified by linking either hydrophobic or hydrophilic groups to the steroid nucleus. The octan-1-ol/water partition coefficient (*log P*), which is a measurement of lipophilicity, has been calculated by the *Virtual log P* method for 15 bile salts derivatives, some of them bearing large hydrophobic groups and other highly hydrophilic substituents at the C3-position of the steroid nucleus. The theoretical partition coefficient and the critical micelle concentration, measured experimentally, are tightly related by a double-logarithm relationship ($Virtual\log P=-\left(1.00\pm 0.09\right)\log\left(\frac{cmc}{\mathrm{mM}}\right)+\left(2.79\pm 0.09\right)$) meaning that the Gibbs free energies for the transfer of a bile anion from water to either a micelle or to octan-1-ol differ by a constant. The equation also means that *cmc* can be used as a measurement of lipophilicity.

A previously published example nicely illustrates the different biological behavior of bile salt derivatives with extreme *log P* values.

## Figures and Tables

**Table 1:** Critical micelle concentration (expressed as molar concentration, *cmc*, and mole fraction, *x_cmc_*), aggregation number, *n*, and thermodynamic parameters for the demicellization of the indicated surfactants at different temperatures.

Surfactant	*cmc/*mM	10^6^*x_cmc_*	*T*/K	*n*	Δ*G_demic_* kJ mol^−1^	Δ*H_demic_* kJ mol^−1^	Δ*S_demic_* J mol^−1^K^−1^
NaAdC	0.323	5.82	283.15	6.16	25.1	−8.2	−118
	0.433	7.81	288.15	6.53	25.2	−6.5	−110
	0.296	5.33	293.15	5.88	25.9	−5.4	−107
	0.336	6.06	298.15	7.38	27.3	−2.9	−101
NaAdCH_2_C	0.488	8.79	288.15	8.56	27.2	−6.4	−117
	0.473	8.53	293.15	10.5	28.5	−5.3	−115
	0.438	7.89	298.15	11.3	28.8	−3.2	−107
	0.442	7.96	303.15	10.3	29.2	−1.5	−101
	0.343	6.18	313.15	11.1	31.0	1.5	−94
	0.378	6.81	318.15	13.2	32.3	2.1	−95
Na*t*ButPhC	0.182	3.28	298.15	48.1	34.4	3.1	−105
	0.220	3.95	303.15	39.3	34.4	5.6	−95
	0.201	3.62	308.15	49.7	35. 4	8.4	−88
	0.220	3.95	313.15	53.4	35.8	10.9	−80
	0.256	4.61	318.15	33.9	35.5	12.0	−74

**Table 2:** log *P* values calculated by the indicated methods for natural unconjugated bile salts, for which experimental values have been measured (Roda et al. [9]).

Compound	*Log P* (iLOGP)	*Log P* (XLOGP3)	*Log P* (WLOGP)	*Log P* (MLOGP)	*Log P* (SILICOS-IT)	Virtual logP	*log P* exp, Roda et al.
NaC, I,	2.84	2.022	2.11	3.05	2.53	1.3768	1.1
NaHC, XIV	3.06	2.8	2.11	3.05	2.53	1.5761	1.84
NaDC, II,	3.07	3.5	3.14	3.88	3.42	2.3453	2.65
NaCheno, VIII	3.21	3.08	3.14	3.88	3.42	2.2348	2.25
NaUDC, XIII	3.14	3.08	3.14	3.88	3.42	2.346	2.2
NaHDC, XV	3.15	2.8	3.14	3.88	3.42	2.2667	2.28
$\sum {\left(\log P_{theor}-\log P_{exp}\right)}^{2}/n$	1.209	0.7046	0.6247	2.470	1.212	0.0435	

## Data Availability

Not applicable.

## Supplementary Materials

The following are available online at https://www.mdpi.com/article/10.3390/ijms22136684/s1.
