# Supplementary material for: Highly Hydrophilic and Lipophilic Derivatives of Bile Salts
Source: Int J Mol Sci. 2021 Jun 22;22(13):6684. doi: 10.3390/ijms22136684 (PMC8268814; doi:10.3390/ijms22136684)
Supplement: Supplementary file 1 [file ijms-22-06684-s001.zip › ijms-1238291-supplementary.pdf]

International Journal of *Molecular Sciences*

MDPI

IJMS-123891

Supplementary Information

Table S1 Structures of the studied compounds

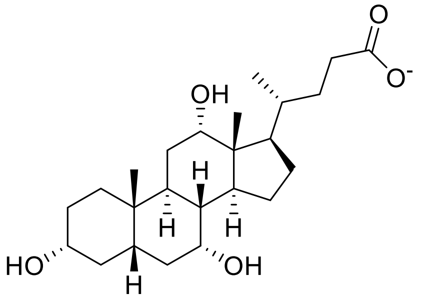 I (NaC) Virtual*logP*=1.3768	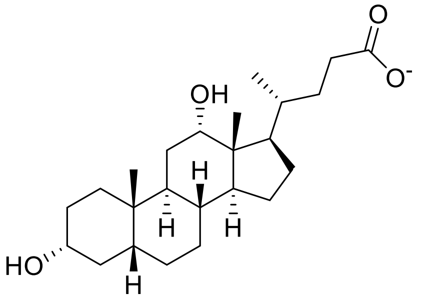 II (NaDC), Virtual*logP*=2.3453
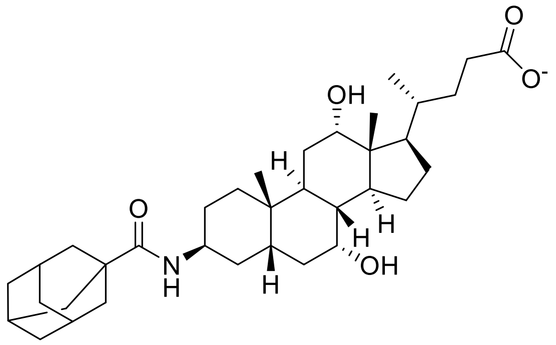 III (NaAdC), Virtual*logP*=3.2291	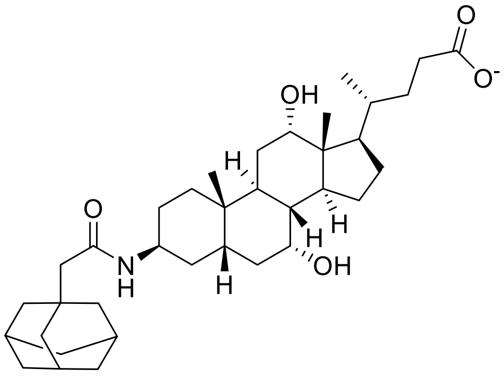 IV (NaAdCH_2C), Virtual*logP*=3.2535
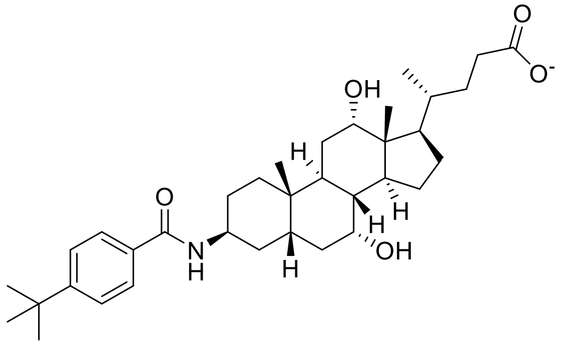 V (Na*t*butPhC), Virtual*logP*=4.2301	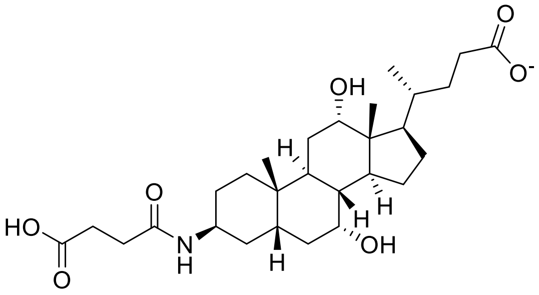 VI (NaSuccC), Virtual*logP*=1.0046
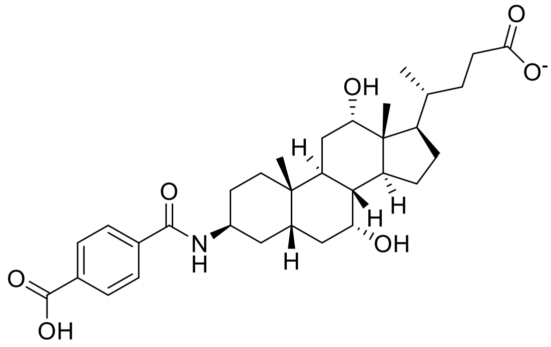 VII, NaterephtC, Virtual*logP*=1.4084	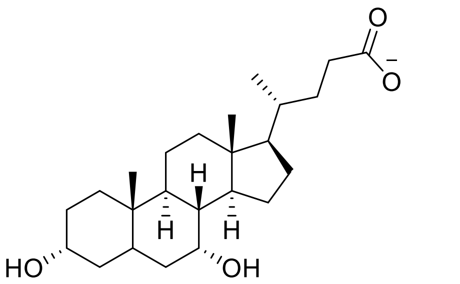 VIII (sodium chenodeoxycholate, NaCDC), Virtual*logP*=2.2348

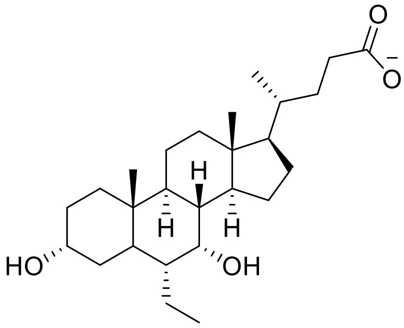 IX (sodium obeticholate), Virtual*logP*=3.0851	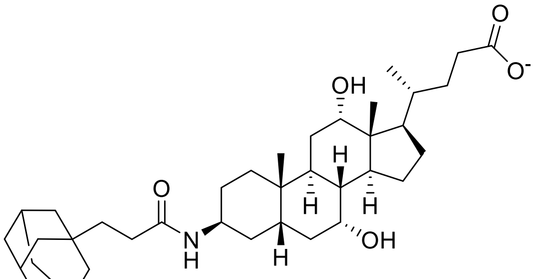 X (NaAd$(CH_2)_2$C), Virtual*logP*=3.8293
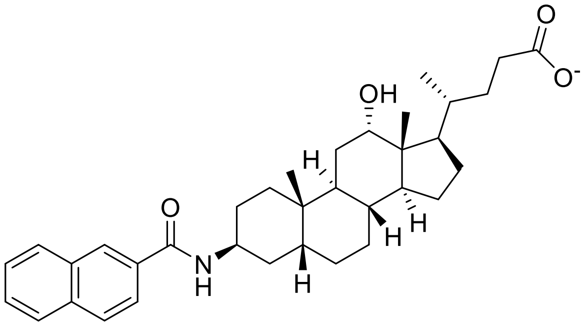 XI (NaNAPhDC), Virtual*logP*=4.4634	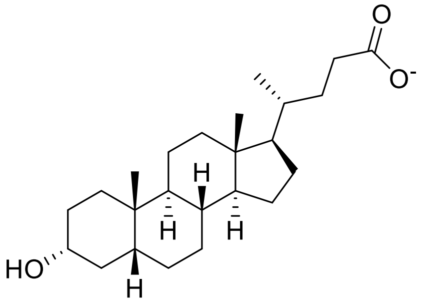 XII (sodium litocholate), Virtual*logP*=3.5081
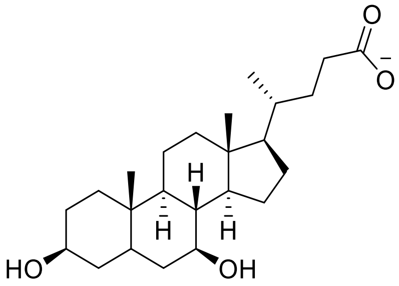 XIII (sodium ursodeoxycholate), Virtual*logP*=2.3460	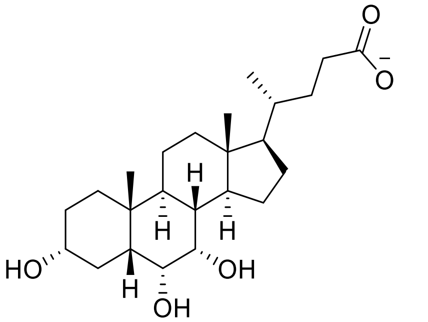 XIV (hyocholate), Virtual*logP*=1.5761
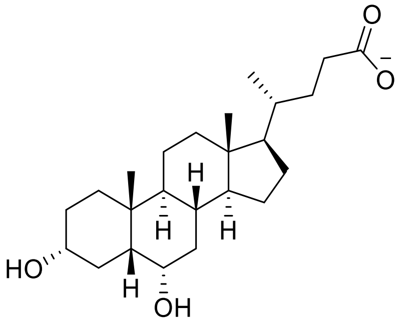 XV (hyodeoxycholate), Virtual*logP*=2.2667	

Surface tension measurements were carried out in a Drop volume Tensiometer TVT2 from Lauda. Temperature was kept constant at 25 °C by recirculating water from a PolyScience 9100 thermostat. Average surface tension values were obtained from four series of 3-6 drops each. Sets of solutions were prepared according to the step-by-step dilution-extraction method [1].

cmc determination of NaNAPhDC

The synthesis was published by Trillo *et al* [2].

Experimental conditions: NaOH/M=0.01.

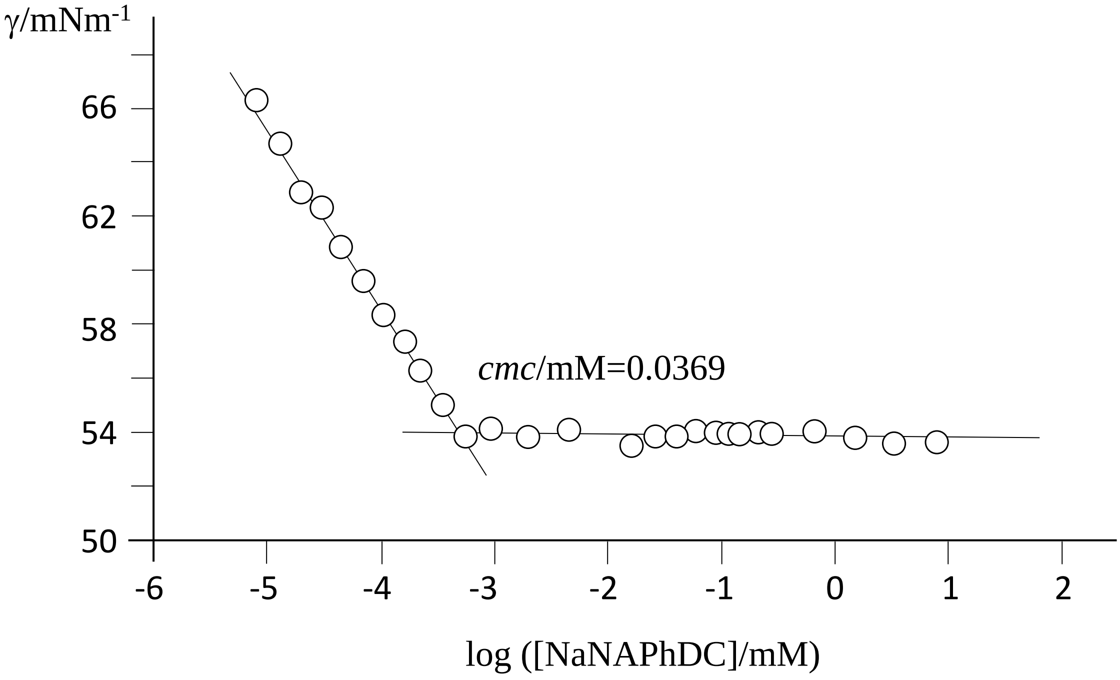

FigureS1. ***cmc* determination of NaNAPhDC** (sodium 4-((3S,5R,8R,9S,10S,12S,13R,14S)-3-(2-naphthamido)-12-hydroxy-10,13-dimethylhexadecahydro-1H-cyclopenta[a]phenanthren-17-yl)pentanoate)

cmc **determination of NaAd(CH_2)$_2$C**:

sodium 4-((3S,5R,8R,9S,10S,12S,13R,14S)-3-(3-((3S,5S,7S)-adamantan-1-yl)propanamido)-12-hydroxy-10,13-dimethylhexadecahydro-1H-cyclopenta[a]phenanthren-17-yl)pentanoate.

The synthesis was published by Miragaya [3].

Experimental conditions: Carbonate/Bicarbonate buffer 50.60 mM, pH 10.35

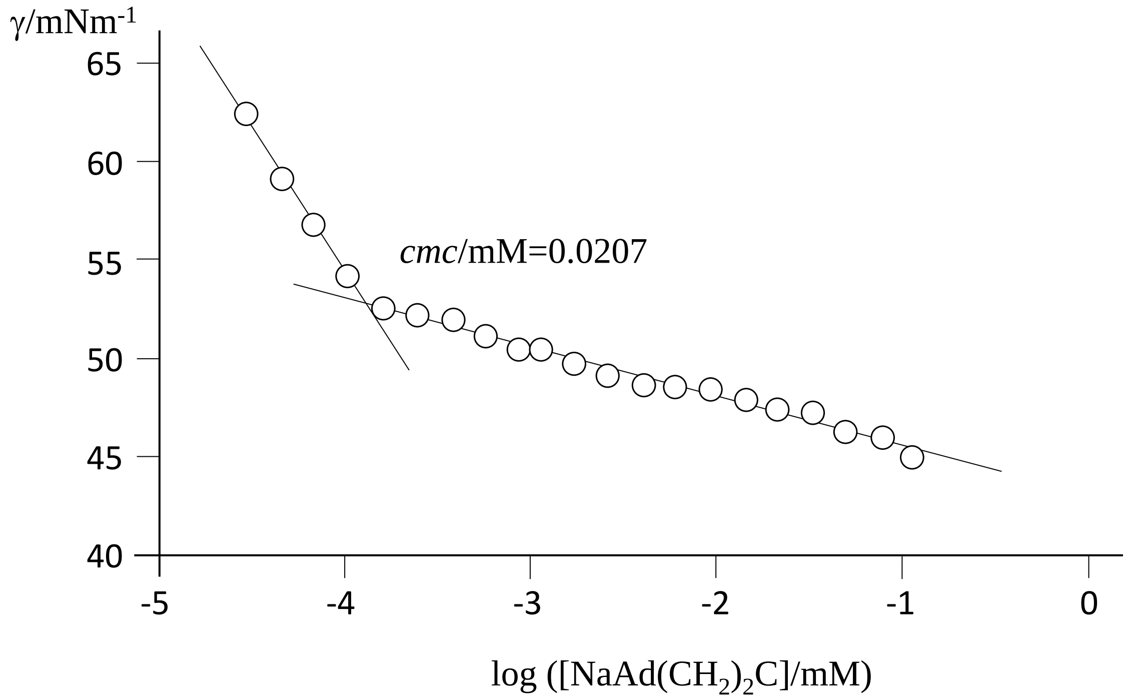

Figure S2. ***cmc*** **determination of NaAd(CH_2)$_2$C**: sodium 4-((3S,5R,8R,9S,10S,12S,13R,14S)-3-(3-((3S,5S,7S)-adamantan-1-yl)propanamido)-12-hydroxy-10,13-dimethylhexadecahydro-1H-cyclopenta[a]phenanthren-17-yl)pentanoate.

1. Gill, S.J.; Dec, S.F.; Olofsson, G.; Wadsö, I. Anomalous heat capacity of hydrophobic solvation. *J. Phys. Chem.* **1985**, *89*, 3758-3761.
2. Trillo, J.V.; Meijide, F.; Jover, A.; Soto, V.H.; de Frutos, S.; di Gregorio, M.C.; Luciano;, G.; Vázquez Tato, J. Self-aggregation mechanism of a naphthylamide cationic derivative of cholic acid. From fibers to tubules. *RSC Adv.* **2014**, *4*, 5598-5606.
3. Miragaya Otero, J. Síntesis y caracterización de surfactantes derivados de sales biliares, adamantanos, norbornanos, tioles, nanopartículas de Au y resinas epoxi. Santiago de Compostela, 2015.
